# [Retracted] *LINC00665* functions as a competitive endogenous RNA to regulate AGTR1 expression by sponging miR‑34a‑5p in glioma

**DOI:** 10.3892/or.2024.8773

**Published:** 2024-07-08

**Authors:** Yongyue Dai, Yucheng Zhang, Maolin Hao, Renwu Zhu

Oncol Rep 45: 1202–1212, 2021; DOI: 10.3892/or.2021.7949

Following the publication of the above paper, it was drawn to the Editor's attention by a concerned reader that the ‘Control’ data panel shown for the EdU assay experiment in Fig. 6D on p. 1209 was strikingly similar to a data panel featured in Fig. 7 that had already been submitted to the journal *Cancer Management and Research* by different authors at different research institutes [Chen T-J, Gao F, Yang T, Li H, Li Y, Ren H and Chen M-W: Knockdown of linc-POU3F3 suppresses the proliferation, apoptosis, and migration resistance of colorectal cancer. Cancer Manag Res 12: 4379–4390, 2020].

Owing to the fact that contentious data in the above article had already been submitted for publication prior to its submission to *Oncology Reports*, the Editor has decided that this paper should be retracted from the Journal. The authors were asked for an explanation to account for these concerns, but the Editorial Office did not receive a reply. The Editor apologizes to the readership for any inconvenience caused.

